# Early treatment of posterior crossbite - a randomised clinical trial

**DOI:** 10.1186/1745-6215-14-20

**Published:** 2013-01-22

**Authors:** Carsten Lippold, Thomas Stamm, Ulrich Meyer, András Végh, Tatjana Moiseenko, Gholamreza Danesh

**Affiliations:** 1 Poliklinik für Kieferorthopädie, Universität Münster, Waldeyerstr. 30, 48149, Münster, Germany; 2 Klinik für Mund und Kiefer-Gesichtschirurgie, Universität Düsseldorf, Moorenstr. 5, 40225, Düsseldorf, Germany; 3 Department of Orofacial Orthopedics and Orthodontics, Heim Pál Children’s Hospital, Ulloi ut 86, Budapest, 1089, Hungary; 4 Fakultät für Gesundheit (Department für Zahn-, Mund- und Kieferheilkunde), Lehrstuhl für Kieferorthopädie, Universität Witten/Herdecke, Alfred-Herrhausen-Str. 50, 58448, Witten, Germany

## Abstract

**Background:**

The aim of this randomised clinical trial was to assess the effect of early orthodontic treatment in contrast to normal growth effects for functional unilateral posterior crossbite in the late deciduous and early mixed dentition by means of three-dimensional digital model analysis.

**Methods:**

This randomised clinical trial was assessed to analyse the orthodontic treatment effects for patients with functional unilateral posterior crossbite in the late deciduous and early mixed dentition using a two-step procedure: initial maxillary expansion followed by a U-bow activator therapy. In the treatment group 31 patients and in the control group 35 patients with a mean age of 7.3 years (SD 2.1) were monitored. The time between the initial assessment (T1) and the follow-up (T2) was one year. The orthodontic analysis was done by a three-dimensional digital model analysis. Using the ‘Digimodel’ software, the orthodontic measurements in the maxilla and mandible and for the midline deviation, the overjet and overbite were recorded.

**Results:**

Significant differences between the control and the therapy group at T2 were detected for the anterior, median and posterior transversal dimensions of the maxilla, the palatal depth, the palatal base arch length, the maxillary arch length and inclination, the midline deviation, the overjet and the overbite.

**Conclusions:**

Orthodontic treatment of a functional unilateral posterior crossbite with a bonded maxillary expansion device followed by U-bow activator therapy in the late deciduous and early mixed dentition is an effective therapeutic method, as evidenced by the results of this RCT. It leads to three-dimensional therapeutically induced maxillary growth effects. Dental occlusion is significantly improved, and the prognosis for normal craniofacial growth is enhanced.

**Trial registration:**

Registration trial DRKS00003497 on DRKS

## Background

In children presenting with a functional unilateral posterior crossbite, the maxillary complex is often constricted [1-3]. This abnormal morphological situation is aetiologically based on a multicausal genetic system [4] and influenced in craniofacial growth by different aetiological factors, such as impaired nasal breathing and muscular dysfunction [5-7], as well as prolonged sucking habits after the second year of life [8,9]. Epidemiological studies vary due to the examined collectives and study criteria, though they reveal a prevalence of between 4% and 16% [10-15]. A functional chain is induced by the maxillary transversal underdevelopment beginning in the deciduous dentition. The interrelation of maxillary and mandibular teeth varies in children between the centric and the maximum intercuspid position. In the centric relation of the condyles with midline concordance, the lower teeth do not occlude in a maximum cuspid-fossa relationship. This unstable maxillomandibular buccal-cuspid occlusion leads to a functional shift of the mandible in maximum occlusion, consequently resulting in a functional unilateral posterior crossbite with midline deviation [6,12,16,17]. In subsequent craniofacial development, a functional unilateral posterior crossbite leads to increased growth on the non-crossbite side and to impairment in the crossbite side [18]. Progredient adaptation of the soft and hard tissues manifests in a unilateral crossbite and possibly results in facial asymmetry [19-21]. However, young children with deciduous or early mixed dentition do not necessarily show signs and symptoms of craniomandibular dysfunction, as this can develop later in growth [22-24].

The literature discusses early orthodontic treatment of functional unilateral posterior crossbites to prevent skeletal manifestations and to improve functional parameters [19,20]. The evidence for treatment effects is in homogeneous due to variations in patient sample size, study protocols and the often-missing control group with the same initial diagnosis [25-29].

### Study aim

The aim of our study was to perform a randomised clinical trial with a control and a therapy group with an identical initial diagnosis: functional unilateral posterior crossbite. A standardised study protocol was used to analyse the effects of early orthodontic treatment of functional unilateral posterior crossbite in children with deciduous or early mixed dentitions.

The general scientific aims were to examine, in detail, the treatment effects of orthodontic interventions in comparison to normal growth effects in the control group in patients presenting functional unilateral posterior crossbite, specifically regarding:

1. the sagittal, vertical and transversal dimensions of the maxilla and mandible

2. the midline deviation between the anterior teeth of the maxilla and mandible

3. the sagittal overjet and vertical overbite.

## Methods

### Study design and blinding

**Figure 1:**
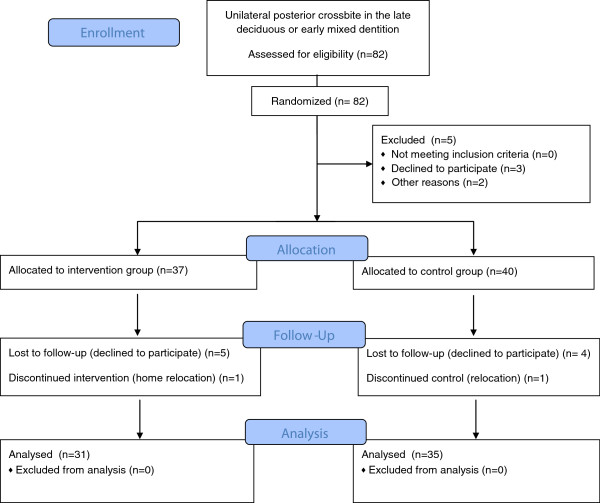
Workflow.

The study was a randomised clinical trial with two different groups: one therapy group and one control group with an identical initial diagnosis of functional unilateral posterior crossbite in the late deciduous or early mixed dentition with no midline deviation during orthodontic treatment, persisting habits, general diseases with permanent medication (for example, diabetes mellitus), syndromes, cleft lip and palate, general impairments and structural orthopaedic diseases. A brief summary of the study workflow according to the criteria of the Consort group [29,30] with measurement events and study arms, as well as the development of sample sizes, is presented in Figure 1. The study protocol, the patient number, the examiner number (two specialists in orthodontics) and calibration for this randomised clinical trial was assessed prior to patient recruiting in close cooperation with the Center for Clinical Trials Münster (the Centre for Clinical Trials Münster is a joint institution of Münster University’s Medical Sciences Division and University Hospital Münster). The study was arranged according to the Helsinki criteria and authorised by the local Ethics Committee of the Medical Faculty, Wesphalian Wilhelms University, Münster (Germany). It was registered by the German register of clinical trials (http://www.drks.de) with the registration number DRKS00003497.

### Patients

From the initial study sample that met the inclusion criteria, the parents of 82 patients signed the informed consent and received a block randomisation with a block length of 20 and an allocation ratio of 1:1 [31]. The patients were divided into a treatment group (40 patients, mean age of 7.3, SD 2.2) and a control group (42 patients, mean age 7.2, SD 2.0). The gender ratio was nearly equal at the beginning of the study. In the intervention group, 37 children received orthodontic treatment according to an early orthodontic treatment concept. For 40 children in the control group, no orthodontic treatment in the observation period was performed. However, these patients received the same orthodontic treatment as did those in the therapy group after the follow-up appointment. The dropout at T1 comprised five patients, who were rejected by the study protocol after randomisation. The interval between the start of treatment (T1) and end of treatment (T2) was 12 months. At T2, a total of 66 patients (mean age 8.3, SD 2.2; 35 control patients, mean age 8.2, SD 2.1) with a nearly equal gender ratio (30 males and 36 females) were examined. Eleven patients dropped out at T2 for the following reasons: two patients interrupted treatment due to personal reasons, four patients stopped during therapy, and five in the control group failed to meet the examination deadline. The data were analysed per protocol.

### Orthodontic treatment

**Figure 2:**
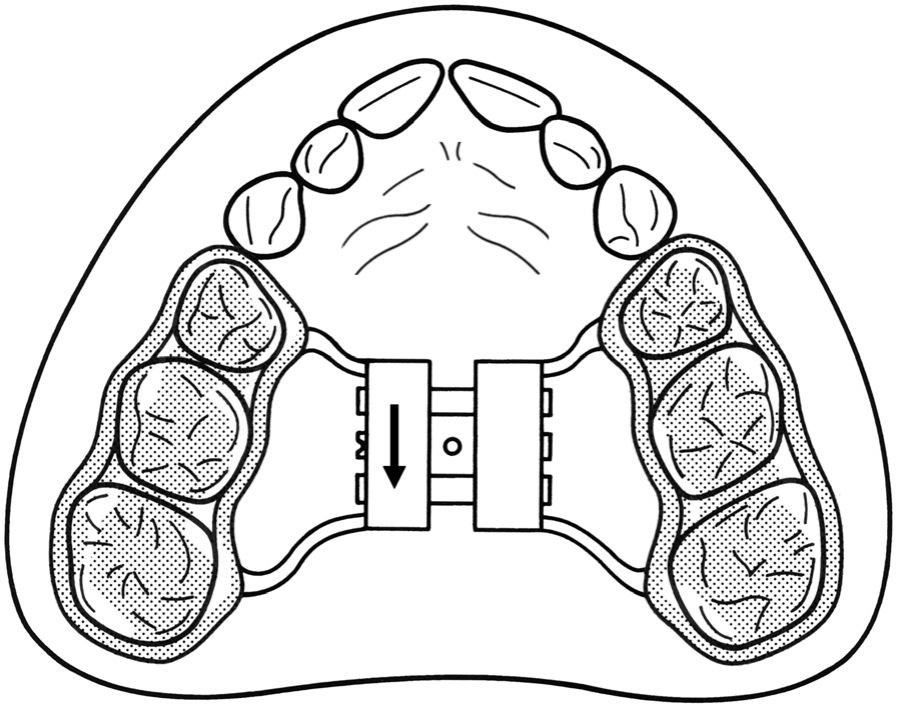
**Bonded maxillary expansion.** Palatal expansion appliance used for slow expansion of the maxillary bones, bonded onto the posterior teeth.

The orthodontic therapy was principally divided into two different steps: 1) the initial maxillary expansion and 2) subsequent activator treatment for midline correction and functional rehabilitation. For the maxillary expansion, a bonded hyrax according to McNamara [32,33] was applied, which initially unlocked the occlusion and was worn 24 hours a day. This orthodontic expansion device (Figure 2) was composed of a wire matrix (1.1 mm) wrapped around the posterior teeth and soldered to the expansion hyrax (Memory Anatomic Expander Type S, spring deflection: 1 mm, spring force: 500 cN, total expansion: 8 mm; Forestadent, Pforzheim, Germany). A resin bite plateau (Palapress clear; Heraeus Kulzer, Hanau, Germany) was polymerised onto the posterior teeth and mechanically bonded to the adjacent wire matrix. The hyrax expansion device was bonded with glass ionomer cement (Ketac Cem; 3M ESPE, Seefeld, Germany). After a clear treatment statement for the patients and their parents, a recommended frequency of once-daily activation was mandatory (that is, each activation resulted in a 0.2 mm daily expansion). The total expansion, including 1 mm of relapse prevention, was calculated by analysing the initial orthodontic plaster models of the patients. An individual protocol was established to fulfil the expansion in approximately three weeks by activating the screw every second day. After the completion of maxillary expansion (mean 3.2 weeks, SD 1.2) came the retention period (mean 12.6 weeks, SD 1.8), resulting in a total time of 16.2 weeks (SD 0.6) for the bonded hyrax *in situ*.

**Figure 3:**
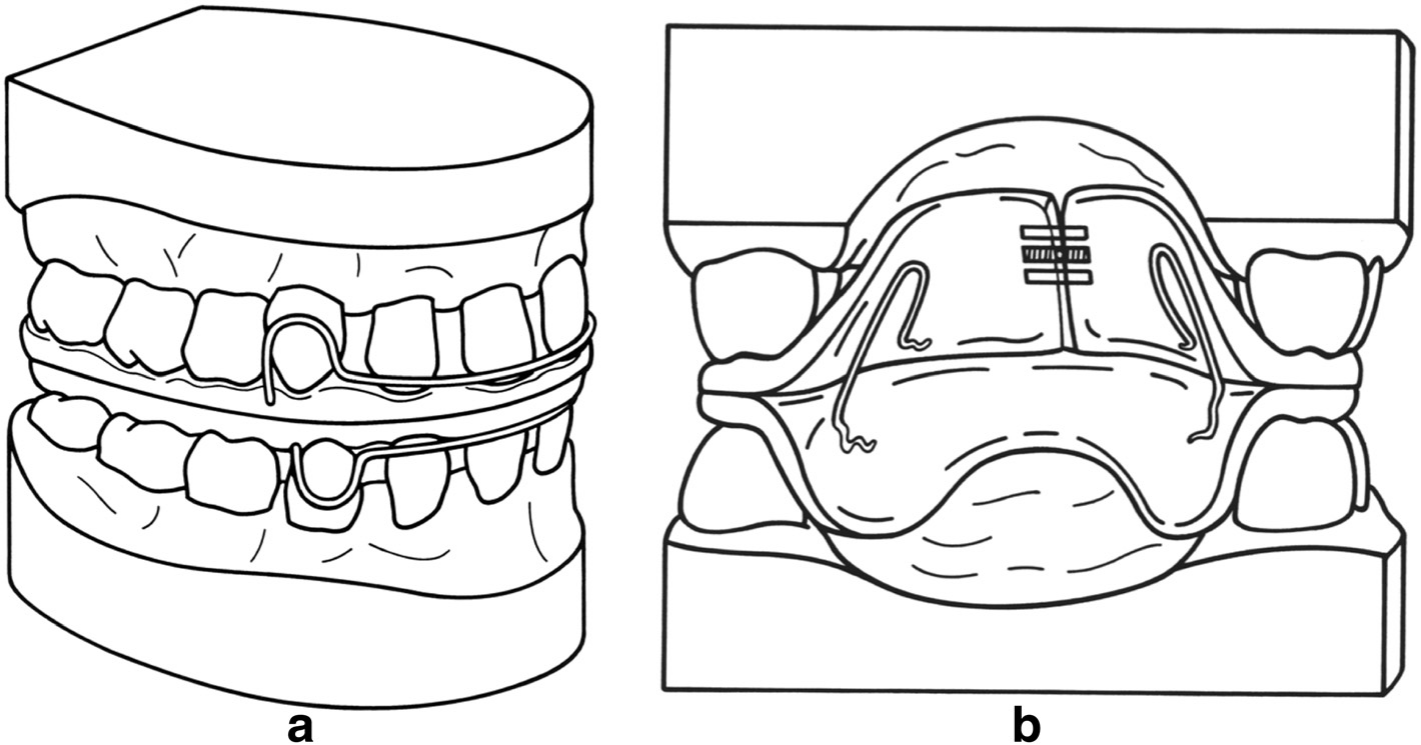
**U-bow activator.** U-bow activator Type 1, as described by Karwetzky, used to achieve midline coordination and retain palatal expansion. **(a)** Outer view, **(b)** inner view.

For retention of the achieved maxillary expansion and for functional midline coordination, a U-bow activator according to Karwetzky (Figures 3a and 3b) was applied [34,35] for 36.8 weeks on average (Table 1). The U-bow activator is a double-plate activator combined with eponymous U-shaped wire bows on each side (Scheu Dental, Iserlohn, Germany). The wire components consist of protrusive and labial bows on the upper and lower jaws. The maxillary plate utilises an additional transversal expansion screw (expansion screw, 7 mm; Forestadent, Pforzheim, Germany) for retention management of the maxillary expansion. Midline correction is achieved by activating the U-bows unilaterally.

**Table 1:** Treatment time for bonded maxillary expansion and U-bow activator therapy

***Time *(*weeks*)**		***Therapy***
		***Total *(*n *= *31*)**
		***Mean *(*SD*)**
*Bonded hyrax*		16.2 (0.6)
	*expansion period* (*n* = *31*)	3.2 (1.2)
	*retention period* (*n* = *31*)	12.6 (1.8)
*U*-*bow activator*		36.8 (5.4)
	*expansion period* (*n* = *3*)	5.9 (3.5)
	*retention period* (*n* = *31*)	36.1 (5.5)

### Measurement procedure

**Figure 4:**
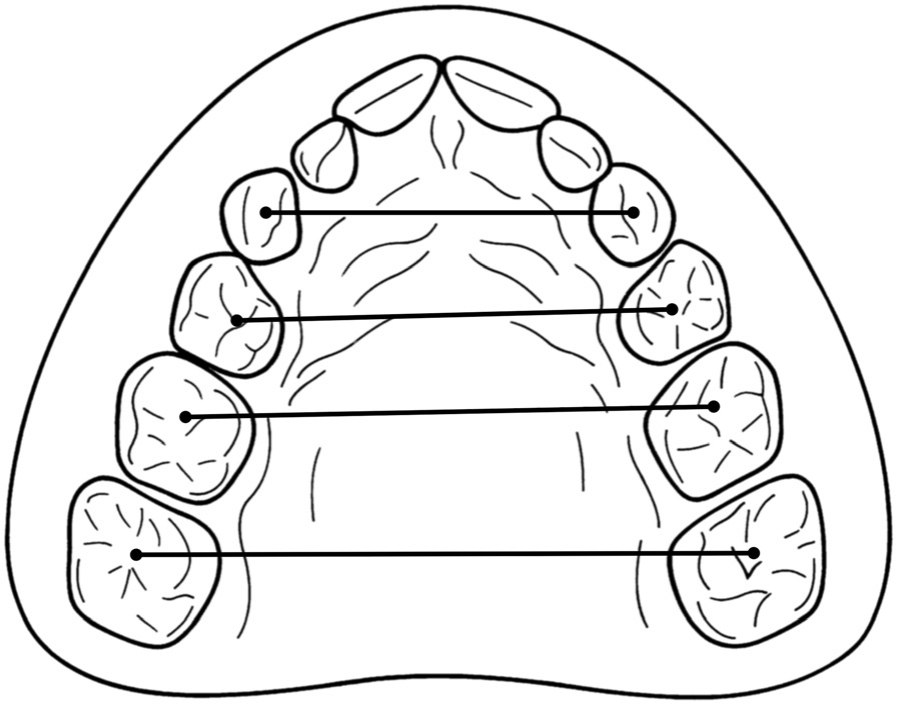
**Maxillary transversal measurements.** For the analysis of the intercanine, anterior, middle and posterior transversal widths, all measurements were performed by projection on the occlusal plane. For the intercanine width, the canine cuspids, the first and second deciduous molars, and the six-year molars, the deepest points of the fossae were taken as reference points.

For the evaluation of orthodontic plaster models at the start (T1) and at the end of the trial (T2), plaster models were fabricated and a cone-beam computed tomography (CBCT)-based digital analysis software ‘Digimodel’ (Orthoproof, Nieuwegein, The Netherlands) was used. All metrical measurements on this mathematical polygon mesh were related to the occlusal plane, which was defined previously. As an output of the digital model analysis data, the following parameters were measured (Table 2):

1. **maxillary transversal measurements** (Figure 4)

**Table 2:** As an output of the digital model analysis data, the following parameters were measured

**Parameter shortcut**	**Unit**	**Parameter**
uitw	[mm]	upper intercanine transversal width (III - III)
uatw	[mm]	upper anterior transversal width (IV - IV)
umtw	[mm]	upper median transversal width (V - V)
uptw	[mm]	upper posterior transversal width (6–6)
malo	[mm]	median arch length on the occlusal plane
aio	[°]	arch inclination on the occlusal plane
apbal	[mm]	anterior palatal base arch length (IV - IV)
mpbal	[mm]	median palatal base arch length (V - V)
ppbal	[mm]	posterior palatal base arch length (6–6)
mapd	[mm]	median anterior palatal depth (IV - IV)
mppd	[mm]	median posterior palatal depth (V - V)
litw	[mm]	lower intercanine transversal width (III - III)
latw	[mm]	lower anterior transversal width (IV - IV)
lmtw	[mm]	lower median transversal width (V - V)
lptw	[mm]	lower posterior transversal width (6–6)
md	[mm]	midline deviation
vob	[mm]	vertical overbite
soj	[mm]	sagittal overjet

For the analysis of the intercanine, anterior, middle and posterior transversal widths, all measurements were performed by projection on the occlusal plane. For the intercanine width, the canine cuspids, the first and second deciduous molars, and the six-year molars, the deepest points of the fossae were taken as reference points.

**Figure 5:**
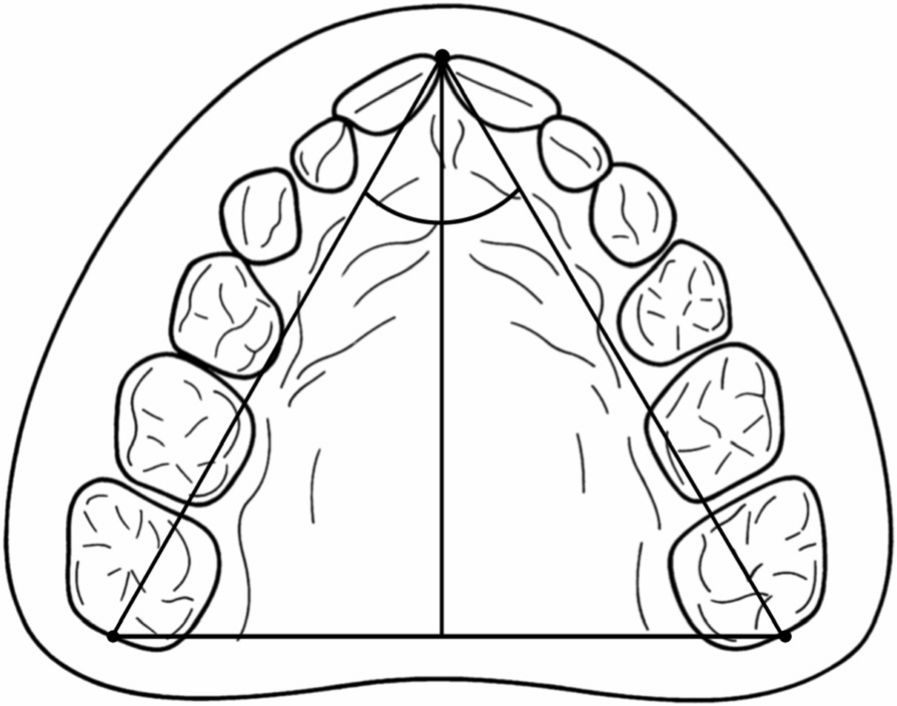
**Maxillary arch length and inclination.** The length of the perpendicular reference line between the tuber plane and the maxillary incisors was defined as the sagittal arch length. The angle that spans between the right and left connecting lines was defined as the arch inclination.

2. **maxillary arch length and inclination** (Figure 5)

The length of the perpendicular reference line between the tuber plane and the maxillary incisors was defined as the sagittal arch length. The angle that spans between the right and left connecting lines was defined as the arch inclination.

**Figure 6:**
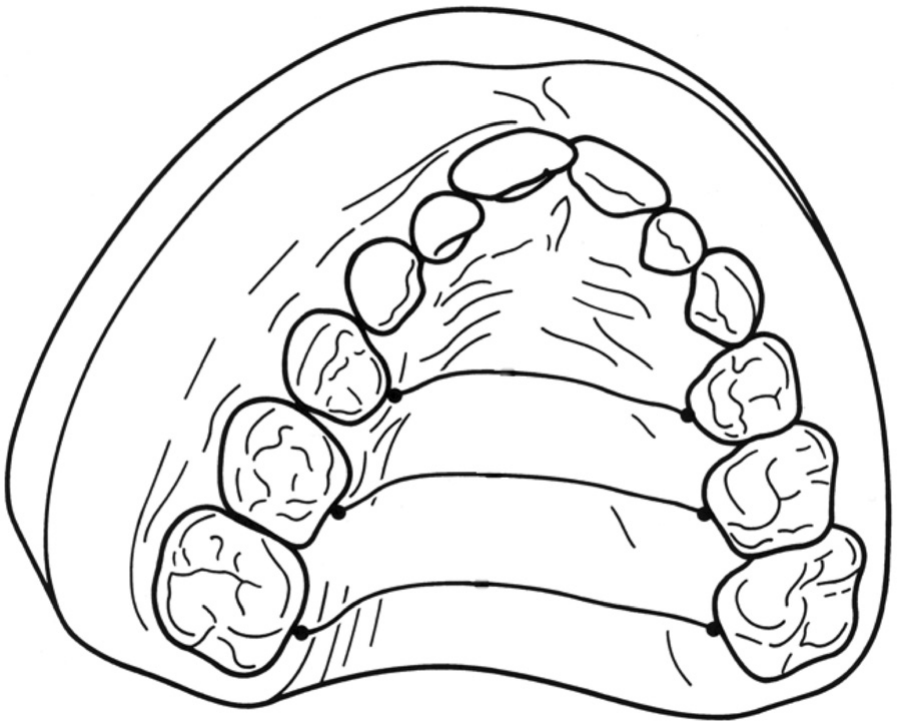
**Transversal palatal base arch length.** Based on the three-dimensional digital model mesh, the anterior, middle and posterior transversal palatal base arch lengths were measured. Reference points were the middle palatal dentogingival transitions of the right and left sides for both the first and second deciduous molars and, if present, for the first permanent molars.

3. **transversal palatal base arch length** (Figure 6)

Based on the three-dimensional digital model mesh, the anterior, middle and posterior transversal palatal base arch lengths were measured. Reference points were the middle palatal dentogingival transitions of the right and left sides for both the first and second deciduous molars and, if present, for the first permanent molars.

**Figure 7:**
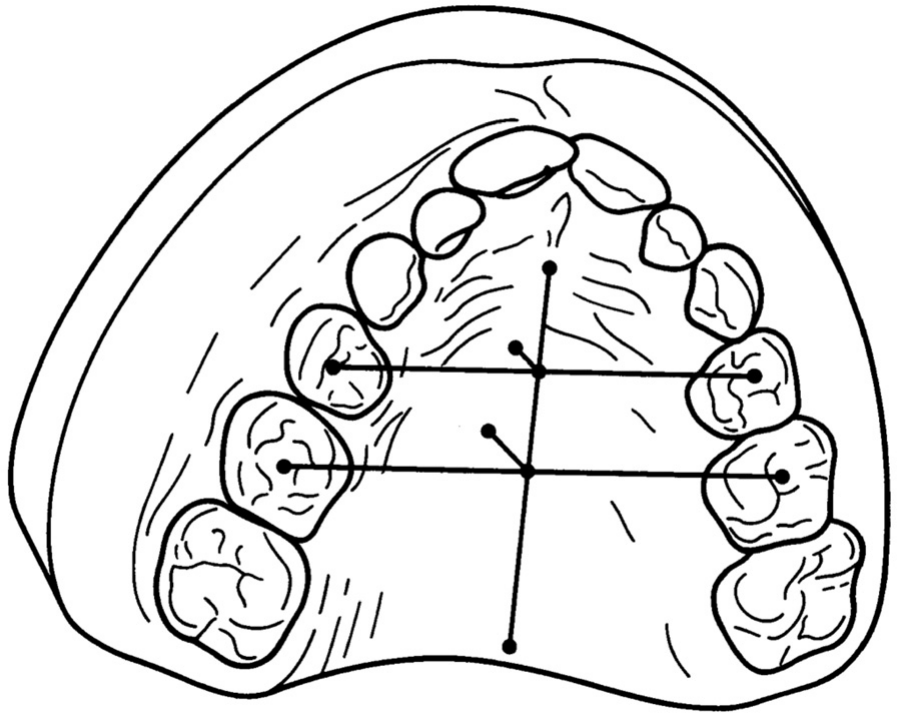
**Palatal depth.** Palatal depth was measured for the first and second deciduous molars perpendicularly to the occlusal plane in the median raphe. Due to the reference point differences between T1 and T2 caused by possible vertical growth of the first permanent molars, no measurement was performed.

4. **palatal depth** (Figure 7)

Palatal depth was measured for the first and second deciduous molars perpendicularly to the occlusal plane in the median raphe. Due to the reference point differences between T1 and T2 caused by possible vertical growth of the first permanent molars, no measurement was performed.

**Figure 8:**
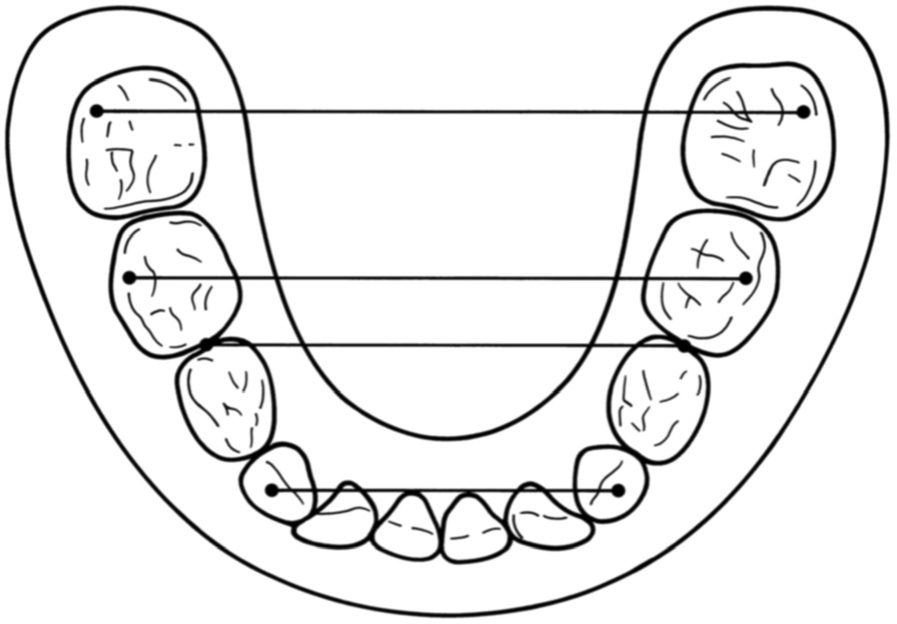
**Mandibular transversal measurements.** The mandibular intercanine distance was measured between the right and left canines. The mandibular anterior transversal width was defined as the distance between the approximal contact points of the mandibular first and second deciduous molars. For the middle and posterior transversal widths, the distance between the distobuccal cusps was registered.

5. **mandibular transversal measurements** (Figure 8)

The mandibular intercanine distance was measured between the right and left canines. The mandibular anterior transversal width was defined as the distance between the approximal contact points of the mandibular first and second deciduous molars. For the middle and posterior transversal widths, the distance between the distobuccal cusps was registered.

**Figure 9:**
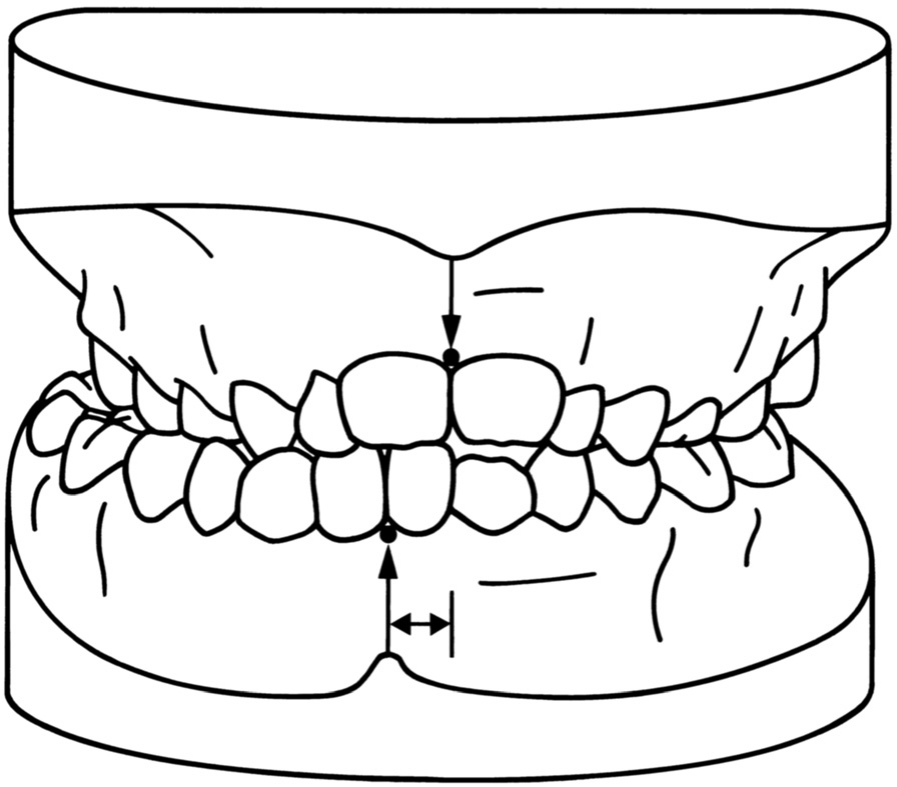
**Midline deviation.** The midline deviation was measured in the frontal plane between the upper and lower midlines on the occlusal plane.

6. **midline deviation** (Figure 9)

The midline deviation was measured in the frontal plane between the upper and lower midlines on the occlusal plane.

**Figure 10:**
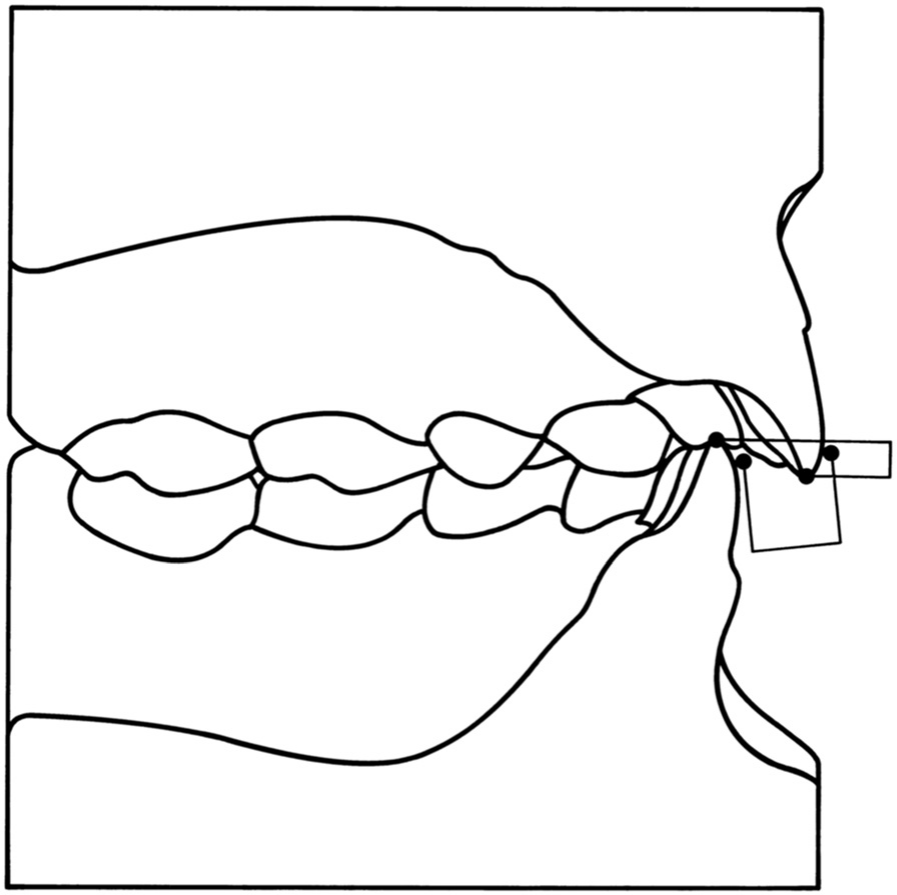
**Overbite and overjet.** The vertical overbite was measured between the edge of the uppermost vertically erupted middle incisor and the corresponding incisal edge of the opposite mandibular tooth perpendicular to the occlusal plane. The sagittal overjet was measured between the most anterior point of the maxillary central incisors and the corresponding reference point on the mandibular incisor.

7. **overbite and overjet** (Figure 10)

The vertical overbite was measured between the edge of the uppermost vertically erupted middle incisor and the corresponding incisal edge of the opposite mandibular tooth perpendicular to the occlusal plane. The sagittal overjet was measured between the most anterior point of the maxillary central incisors and the corresponding reference point on the mandibular incisor.

### Statistics

The advice and planning for the statistical analysis was performed in close cooperation with the Coordinating Centre for Clinical Trials (KKS Network, Münster, Germany).

SPSS 12.0 (Lead Tech., Chicago, IL, USA) was used for the statistical analysis of the registered variables. In regard to the descriptive statistics, mean values and standard deviations were indicated. The initial groups (therapy and controls) were compared with the Kolmogorow-Smirnow test at T1 for deviations from normal distribution. No significant deviations were obtained; therefore, the *t* test was used for the analysis of significant differences for all pair-wise comparisons.

The significance levels were set as follows: *P* <0.001*** ‘very significant’, *P* <0.01** ‘highly significant’, and *P* <0.05* ‘significant’.

To analyse the error of the method, a repeated measurement for 10 randomly chosen digital models with anonymisation was performed at a weekly interval. The error was assessed using Dahlberg’s formula: s = √(×1-×2)2/2. The error levels were set at 0.5° and 0.5 mm according to Trpkova *et al*. [36]. The standard errors were below 0.5 mm and 0.5° for all measured variables.

## Results

Measurements at T1 and T2 for the control and therapy groups are presented in Table 3. Transversal maxillary expansion was statistically very significant in the therapy group between T1 and T2 for the intercanine distance, the anterior, median and posterior transversal widths. For the control group, a very significant growth effect was detected for the intercanine distance and the anterior transversal width between T1 and T2. No significant differences were measured for the median and posterior transversal widths. The difference between orthodontic treatment effects in the therapy group and normal maxillary growth in the control group at T2 was very significant. Statistically, a very significant difference for the intercanine distance, the anterior, median and posterior transversal widths were observed between the therapy group and the control group.

**Table 3:** **Results of the statistical analysis (median, SD and *t *test)**

			**Control**			**Therapy**			**Control-therapy (T2)**
**Code**	**Unit**	**Parameter**		**T1**	**T2**	***t *test**	**T1**	**T2**	***t *test**	***t *test**
uitw	[mm]	upper intercanine transversal width (III - III)		27.9 (2.2)	28.9 (2.2)	0.000***	29.0 (2.6)	32.6 (2.7)	0.000***	0.000***
uatw	[mm]	upper anterior transversal width (IV - IV)		32.2 (1.9)	32.7 (2.0)	0.006**	32.6 (2.2)	36.7 (2.8)	0.000***	0.000***
umtw	[mm]	upper median transversal width (V - V)		37.2 (2.4)	37.7 (2.3)	n.s.	36.8 (1.8)	41.7 (3.1)	0.000***	0.000***
uptw	[mm]	upper posterior transversal width (6–6)		42.6 (3.1)	43.4 (2.3)	n.s.	42.2 (2.6)	47.3 (2.5)	0.000***	0.000***
malo	[mm]	median arch length on the occlusal plane		34.0 (4.9)	34.7 (4.6)	n.s.	34.8 (4.7)	34.4 (4.8)	n.s.	0.045*
aio	[°]	arch inclination on the occlusal plane		66.1 (5.5)	66.9 (7.3)	n.s.	65.6 (4.5)	70.8 (5.4)	0.000***	0.001**
apbal	[mm]	anterior palatal base arch length (IV - IV)		34.7 (4.1)	34.6 (4.0)	n.s.	35.7 (3.4)	38.8 (3.9)	0.000***	0.000***
mpbal	[mm]	median palatal base arch length (V - V)		38.5 (4.2)	39.4 (3.9)	0.006**	40.4 (3.5)	44.3 (3.9)	0.000***	0.000***
ppbal	[mm]	posterior palatal base arch length (6–6)		41.2 (2.9)	41.6 (2.9)	n.s.	41.5 (4.1)	46.1 (3.8)	0.000***	0.000***
mapd	[mm]	median anterior palatal depth (IV - IV)		11.4 (1.6)	11.5 (2.0)	n.s.	12.0 (1.8)	11.1 (1.8)	0.001**	0.002**
mppd	[mm]	median posterior palatal depth (V - V)		13.7 (1.7)	14.0 (1.8)	n.s.	14.6 (2.1)	14.2 (2.1)	n.s.	0.011*
litw	[mm]	lower intercanine transversal width (III - III)		25.2 (1.7)	25.4 (1.6)	n.s.	25.8 (1.9)	25.9 (1.7)	n.s.	n.s.
latw	[mm]	lower anterior transversal width (IV - IV)		33.4 (1.6)	33.6 (1.9)	n.s.	33.8 (1.8)	34.2 (1.6)	n.s.	n.s.
lmtw	[mm]	lower median transversal width (V - V)		41.3 (2.4)	41.4 (2.0)	n.s.	41.6 (2.0)	41.8 (1.9)	n.s.	n.s.
lptw	[mm]	lower posterior transversal width (6–6)		47.2 (2.5)	47.7 (2.6)	n.s.	48.1 (2.0)	48.6 (1.7)	n.s.	n.s.
md	[mm]	midline deviation		1.9 (1.2)	2.1 (1.3)	n.s.	2.1 (1.3)	0.5 (0.5)	0.000***	0.000***
vob	[mm]	vertical overbite		0.7 (1.9)	1.0 (1.8)	n.s.	0.3 (2.4)	1.8 (2.1)	0.000***	0.005**
soj	[mm]	sagittal overjet		3.2 (2.0)	3.4 (2.4)	n.s.	3.6 (1.5)	3.3 (1.2)	n.s.	n.s.

*P* <0.001*** ‘very significant’, *P* <0.01** ‘highly significant’, *P* <0.05* ‘significant’, n.s. ‘not significant’.

Regarding the sagittal maxillary arch, the length in projection on the occlusal plane remained stable in the control group, although this decreased comparatively significantly in the therapy group.

The maxillary arch inclination increased very significantly for the therapy group between T1 and T2 but showed no significant differences for the control group. The t-test revealed a highly significant difference between the therapy and control groups at T2.

The transversal basal arch length in the anterior, middle and posterior regions indicated a statistically very significant increase between T1 and T2 in the therapy group. The control group showed a highly significant difference between T1 and T2 for the median palatal base arch length. The basal arch length increase in the therapy group was very significant compared to that in the control group in all three measured regions: anterior, middle and posterior basal arch lengths.

The palatal depth of the first deciduous molars was highly significantly reduced in the therapy group. In contrast, the control group did not show significant changes between T1 and T2. The difference between the control and therapy groups at T2 was determined to be highly significant. In the second deciduous molar region, a slight but not statistically significant increase was detected in the control group. The therapy group showed an insignificantly slight decrease in palatal depths. However, the difference at T2 between the therapy and control groups was statistically significant.

The intercanine, anterior, middle and posterior transversal distances in the mandible revealed neither any statistically significant differences between T1 and T2 for the either the therapy or control group nor intergroup differences at T2.

At T1, the patients in both the therapy and the control groups showed approximately 2 mm of midline deviation. At T2, the midline deviation was very significantly reduced for the therapy group while it was slightly increased for the control group. The therapeutic effect of midline correction was statistically very significant between the therapy and control groups.

The vertical overbite between T1 and T2 remained stable for the control group but increased very significantly for the therapy group. At T2, the deepening of the bite was highly significant between the therapy and control groups. The sagittal overjet, however, showed no statistically significant effects.

## Discussion

In orthodontics, there is a lack of evidence regarding the effects of early treatment of a functional unilateral posterior crossbite in the deciduous and/or early mixed dentitions [26]. Harrison and Ashby [19] focused on a need for randomised clinical trials for posterior crossbites in children. The review for the ‘Cochrane collaboration’ postulates a structured treatment protocol, informed consent and compliance with the Helsinki criteria. Randomised clinical trials in orthodontics should aim to differentiate the impact of orthodontic treatment from natural growth effects and possible self-healing tendencies [25,27-29,37]. Petren and Bondermark [38] were among the first authors to establish a randomised clinical trial for unilateral posterior crossbite correction with an untreated control group. The patients were allocated to four different groups: therapy group using quad-helix, therapy using removable expansion plate, therapy using composite onlay and a control group. However, these authors did not use bonded acrylic expansion plates according to McNamara [32,33] as we used in our present study. The expansion effects were similar in the study of Petren and Bondermark to our study regarding the transversal maxillary expansion.

In our own present study, the protocol was based on the requirements of randomised clinical trials according to the ‘Consort Statement’ developed by the Standards of Reporting Trials (SORT) Group [29,30]. To confirm with the requirements of the local ethics committee, the same standardised orthodontic treatment protocol in the control group was performed after completion of the study.

The analysis of the plaster models is a standard method in orthodontic practice and studies. Based on a three-dimensional computer mesh, this new variable was used in the present study to determine the transversal palatal base arch lengths (Figure 6). This new development enabled a structural analysis of the palatal morphology before and after maxillary expansion. The computerised analysis of plaster models is based on high methodological accuracy [39] and was used in the present study, as it is likely a practical and effective scientific tool for data analysis [40].

The effects of orthodontic treatment in our study were differentiated from normally occurring craniofacial growth because of a randomised established control group. Normal growth in the control group led to an increase in the intercanine width and the anterior transversal width. Such growth effects, however, were too small to compensate the transversal constriction of the maxilla, which was typical in all unilateral crossbite patients. The parameters for the therapy group showed a statistically very significant improvement of the transversal discrepancy in the maxilla for intercanine distance, as well as anterior, middle and posterior transversal widths after orthodontic treatment. In connection with the very significant increase in the arch inclination and the significant decrease in the palatal height in the region of the first deciduous molars, further measurements showed us the development of the maxillary morphology after maxillary expansion: the transversal basal arch length. This parameter showed a highly significant increase in the anterior, middle and posterior regions. Primozic *et al*. [40] detected a statistically significant palatal volume increase as the result of early orthodontic treatment in a group of crossbite patients (mean age 4.9, SD 0.98 years). Combining these findings, we can conclude that the shape of the maxilla is changed by orthodontic treatment to a wider base with less transverse constriction. This may possibly lead to better orofacial muscular function, especially regarding the form and function of the tongue. The tongue can rest at the palatal base and possibly leads to improved morphological development. Because craniofacial growth is influenced by muscular function, the resting position for the tongue after early treatment of a functional unilateral posterior crossbite seems to be an important aetiological factor for long-term stability and normal growth conditions. The timing of treatment seemed to be linked to this muscular dysfunction. Lindner [41] showed that delayed start of treatment in functional unilateral posterior crossbite leads to a prolongation of the treatment time and an increase in orthodontic treatment complexity.

The results of our study cannot be directly transferred to the data of other publications because of variations in the study design: different patient groups regarding age and number of patients, orthodontic treatment methods and statistical analysis. Primozic *et al*. [40] showed in a randomised clinical trial the effects of early orthodontic treatment on palatal volume increase. In their study, a control group with non-crossbite was used. All patients with crossbite were treated with a maxillary expansion device, comparable to the one used in our present study. The randomised clinical trial by Petren and Bondermark showed the successful use of a quad-helix appliance for expansion in the mixed dentition for patients with unilateral posterior crossbite. They used a randomisation of patients with unilateral crossbite into four groups: quad-helix, expansion plate, composite inlay and untreated control group. A comparable study by Thilander *et al*. [11] evaluated different treatment methods such as grinding and application of expansion plates for the treatment of a functional unilateral posterior crossbite in the deciduous dentition. As a control group, children of identical age with normal buccal occlusion were used, although no randomisation was performed between the groups. However, the positive effect of early interceptive treatment for dentoalveolar development was demonstrated by the results of this study. Geran *et al*. [37] performed a prospective study in the mixed dentition using a bonded acrylic splint rapid maxillary expansion device [32] to assess treatment effects in comparison to those in a control group without malocclusion. Other studies using a bonded palatal expansion device, as was used in our study [25], were based on a different initial diagnosis and a higher mean age of the patients. The time interval between initial recordings and post-treatment controls varies in the described studies from two years [25] to five years [37]. Our randomised clinical trial on functional unilateral posterior crossbite in the late deciduous and early mixed dentition showed the clinical efficacy of an early orthodontic treatment protocol in a one-year period. This is in conclusion to Petren and Bondermark [38].

## Conclusion

Orthodontic treatment of a functional unilateral posterior crossbite with a bonded maxillary expansion device followed by U-bow activator therapy in the late deciduous and early mixed dentition is an effective therapeutic method, as evidenced by the results of this RCT. It leads to three-dimensional therapeutically induced maxillary growth effects. Dental occlusion is significantly improved, and the prognosis for normal craniofacial growth is enhanced.

## Competing interests

The authors declare that they have no competing interests.

## Authors’ contributions

CL, TS, UM, and GD organised the study design, ethical approval, literature review and drafting of the manuscript for this randomised control trial. AV and TM organised the statistical data transfer, data analysis and three-dimensional digital model analysis as well as assisted in the manuscript work for this project. GD and UM performed the statistical analysis and reviewed the study. All authors read and approved the final manuscript.
